# CAR-T in the Treatment of Solid Tumors—A Review of Current Research and Future Perspectives

**DOI:** 10.3390/ijms26199486

**Published:** 2025-09-28

**Authors:** Natalia Picheta, Julia Piekarz, Karolina Daniłowska, Katarzyna Szklener, Sławomir Mańdziuk

**Affiliations:** 1 Student Academic Group, Department of Clinical Oncology and Chemotherapy, Medical University, 20-090 Lublin, Poland; natalia.picheta2812@gmail.com (N.P.); piekarzjulia1@gmail.com (J.P.); karolina02812@gmail.com (K.D.); 2 Department of Clinical Oncology and Chemotherapy, Medical University, 20-090 Lublin, Poland

**Keywords:** CAR-T, chimeric antigen receptor T-cells, solid tumors CAR-T, CAR-T limitations

## Abstract

The aim of this narrative review is to present the current state of knowledge regarding the use of chimeric antigen receptor T-cell (CAR-T) therapy in solid tumors. Phase I clinical trials and side effects are discussed. The review is based on an analysis of available scientific publications, primarily phase I trials, Food and Drug Administration (FDA) reports, and PubMed, Scopus, and Google Scholar sources. It includes clinical trials and review articles from 2016 to 2025. Accumulated data indicate promising efficacy of CAR-T therapy in the treatment of certain solid tumors, particularly those of the gastrointestinal tract, although clinical responses were often limited to disease stabilization. The therapy was generally well tolerated, with a low incidence of serious adverse events. Efficacy was found to depend on factors such as the type of target antigen, the presence of conditioning therapy, and the ability to overcome the immunosuppressive tumor microenvironment. CAR-T therapy remains experimental outside of hematological malignancies, but further development, refinement of receptor design, and the search for better molecular targets may make it an effective treatment option for solid tumors as well. Current studies are in early phase and require confirmation in larger-scale randomized trials.

## 1. Introduction

Cancer immunotherapy has revolutionized the approach to cancer treatment in recent years, introducing new therapeutic strategies such as checkpoint inhibitors, monoclonal antibodies, and adaptive therapies. One of the most groundbreaking advancements in this field is chimeric antigen receptor T-cell (CAR-T) therapy—a method that genetically modifies a patient’s own T lymphocytes to give them the ability to recognize and destroy cancer cells [1]. By using a synthetic chimeric antigen receptor (CAR), these cells can bypass the tumor’s immune evasion mechanisms, making this therapy a highly specific and targeted treatment tool.

CAR-T has found its first clinical applications in the treatment of hematological malignancies that are resistant to standard therapies, such as acute lymphoblastic leukemia (ALL), diffuse large B-cell lymphoma (DLBCL), and multiple myeloma [2]. In 2017, the U.S. Food and Drug Administration (FDA) approved the first CAR-T product, tisagenlecleucel, for the treatment of children and young adults with relapsed ALL. Since then, several other products have been approved, including Yescarta, Breyanzi, Abecma, and Carvykti. The European Medicines Agency (EMA) has also approved CAR-T for use in specific indications. These therapies have established a new standard of care for patients who have failed other options [3]. Despite spectacular successes in hematology, the use of CAR-T in solid tumors remains experimental. Solid tumors, such as pancreatic, ovarian, and gastric cancer, are characterized by greater heterogeneity, the presence of an immunosuppressive microenvironment, and difficulties in reaching tumor tissue by T lymphocytes [4]. However, numerous clinical trials are underway to overcome these barriers by improving the design of CAR receptors, using conditioning therapies, and identifying new molecular targets.

The purpose of this narrative review is to summarize the current state of knowledge on CAR-T therapy, with a particular emphasis on its application outside of hematology. We discuss the mechanisms of CAR-T cell action, their design, generations, and clinical data from phase I trials conducted in patients with solid tumors. The review also aims to identify the current limitations and challenges facing CAR-T therapy, as well as potential directions for its future development.

## 2. Materials and Methods

The literature search was conducted to identify clinical studies evaluating the use of CAR-T therapy in solid tumors. The keywords used for database exploration included: “CAR-T”, “chimeric antigen receptor T-cells”, “solid tumors CAR-T”, “CAR-T limitations”. The search was carried out in the timeframe 2014–2025, using the databases PubMed, Scopus, and Google Scholar.

The initial search returned 4723 articles, of which 1542 were from PubMed, 1450 from Scopus, and 1731 from Google Scholar. After removing duplicates and screening for relevance, only full-text articles were considered. A total of 61 papers were reviewed in detail.

Inclusion criteria: original research articles, human studies—clinical trials, reviews and original paper, articles published in English, studies evaluating CAR-T therapy in solid tumors Exclusion criteria: preclinical or animal-only studies, conference abstracts and editorials, articles not available in full text, studies focused exclusively on hematologic malignancies

The final selection included clinical trials phase I investigating CAR-T therapy in solid tumors such as pancreatic cancer, gastric cancer, biliary tract cancer, hepatocellular carcinoma, mesothelioma, and others. The review followed a narrative synthesis approach, summarizing key findings regarding safety, efficacy, therapeutic targets, and clinical outcomes.

## 3. Results

### 3.1. CAR-T Therapy

**Figure 1:**
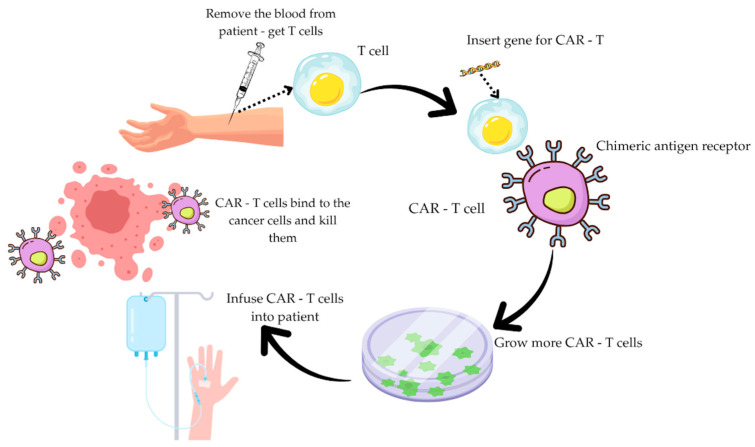
Diagram showing CAR-T cell therapy [4].

CAR-T is a revolutionary therapy that has transformed cancer treatment. CARs are synthetic transmembrane receptors displayed on genetically modified T lymphocytes that recognize specific surface antigens on target cells and eliminate them [5]. It involves collecting T cells from the patient, in vitro activation, genetic modification, and expansion, after which the modified lymphocytes are reintroduced into the patient’s bloodstream. Figure 1 shows a diagram of CAR-T cell therapy.

This process begins with the isolation of T lymphocytes from peripheral blood. Next, a gene encoding CAR is introduced into their genome, most often using viral vectors—retroviral or lentiviral—or gene-editing methods such as clustered regularly interspaced short palindromic repeats (CRISPR) or CRISPR-associated protein 9 (Cas9) [6]. Expression of the transgene leads to the production of a receptor that combines an extracellular antigen-recognition domain, usually derived from a single variable chain antibody fragment (scFv), with intracellular signaling domains—CD3ζ and costimulatory domains—CD28 or 4-1BB. This allows CAR-T lymphocytes to recognize tumor antigens independently of MHC presentation, resulting in their activation and cytotoxic effect on tumor cells [7]. Following transduction, the lymphocytes are expanded in vitro and infused into the patient.

In recent years, numerous modifications have been developed to increase the efficacy and safety of this therapy. These include the introduction of additional genes encoding cytokines that support lymphocyte proliferation and survival (IL-7, IL-15), constructs containing costimulatory ligands, as well as the use of “suicide genes” and pharmacological ON/OFF switches to control CAR-T cell activity [8]. Additionally, precise genome editing techniques such as CRISPR/Cas9 allow for targeted insertion of the transgene at a specific locus, which promotes uniform CAR expression and improved effector cell function.

CAR receptors consist of four functional regions. The first is the extracellular domain, responsible for recognizing and binding the target antigen; it is typically based on a scFv or the variable domain of an antibody heavy chain (VHH) [9]. The second component is the hinge region, which provides the receptor with structural flexibility. The third part is the transmembrane domain, composed of proteins such as CD28, CD8α, CD4, or CD3ζ, whose role is to connect the extracellular and intracellular components and stabilize the entire receptor. The final, key segment is the endodomain, responsible for signal initiation within the cell. This activation most often occurs via the CD3ζ chain, which contains Immunoreceptor Tyrosine-based Activation Motifs (ITAMs), characteristic of the T receptor–CD3 target receptor (TCR/CD3) complex [10].

Currently, CAR receptors are divided into five generations, differing in structure and functionality. First-generation receptors contain only two basic elements: an antigen recognition domain and the CD3ζ signaling domain responsible for T cell activation. The second generation has been improved by the addition of a single costimulatory domain, most often CD28 or 4-1BB, which significantly improves the cells’ ability to survive and proliferate [11]. In the third generation receptors, additional signaling molecules, such as CD40 or OX40, are used in addition to the already present CD28 or 4-1BB, which translates into even stronger proliferation and effector activity of T lymphocytes [12]. The fourth generation, also known as T cells Redirected for Antigen-Unrestricted Cytokine-initiated Killing (TRUCK), is designed to secrete immunomodulatory cytokines such as IL-12 or IL-15 upon antigen recognition, enabling the recruitment of other immune cells to the site of action. The fifth generation, referred to as advanced, may contain complex signaling systems combining multiple co-stimulatory domains, transcription activators, or so-called “logic switches,” aimed at increasing the precision, efficacy, and safety of therapy [13].

### 3.2. Mechanism of Action of CAR-T Cells

To effectively combat cancer cells, CAR-T cells recognize and bind to a specific antigen present on the surface of the target cell. This binding initiates the formation of an immunological synapse (IS), a specially organized molecular structure that enables effective communication between the effector cell and the target [14]. The IS is composed of several layers of supramolecular activation clusters (SMACs) that perform various functions. The central SMAC contains the T cell receptor (TCR) and Lck kinase, which are crucial for the initiation and modulation of T cell activation signaling, as well as its proper termination [15,16]. The surrounding central peripheral SMAC is composed of adhesion molecules, primarily integrins such as lymphocyte function-associated antigen-1 (LFA-1), which stabilize cell to cell contact. The outer layer, the so-called distal SMAC, contains the proteins CD43 and CD45, which regulate the spatial organization of the synapse and signaling [11,13]. Once the immunological synapse is formed, CAR-T cells initiate their cytotoxic effects within minutes through various molecular mechanisms that lead to the elimination of the tumor cell. One of the main mechanisms is the perforin-granzyme pathway, used by cytotoxic T lymphocytes (CTLs) to induce target cell death [17]. CTL granulocytes contain granules filled with perforin and granzymes. After initiating the immunological synapse, the granules migrate to the site of contact, where they fuse with the tumor cell membrane and release perforin. Perforin forms oligomeric channels in the target cell membrane, allowing granzymes to penetrate the cell and induce apoptotic death, through both caspase-dependent and -independent mechanisms [18].

Another important cytotoxic mechanism utilized by CAR-T cells is the Fas-dependent cell death pathway and the Fas ligand (FasL) interaction. Fas (CD95) is a membrane receptor with a death domain (DD) that, upon binding to FasL, also a membrane protein, initiates a signaling cascade leading to apoptosis [19]. Fas binding to FasL recruits the adaptor protein fas-associated death domain protein (FADD), which then binds procaspase-8, leading to the formation of the death-inducing signaling complex (DISC) [20]. Activation of this complex initiates the activation of effector caspases, primarily caspase-3, which carry out organized cell death.

In addition to directly destroying cancer cells, CAR-T cells exert immunomodulatory effects by secreting various cytokines, such as interferon gamma (IFN-γ) and tumor necrosis factor alpha (TNF-α) [21]. These cytokines enhance the immune response, increase CAR-T cell proliferation and activity, and also influence the tumor microenvironment (TME), altering its character. For example, IFN-γ can increase receptor expression on stromal cells, which promotes their sensitization to the immune system. Furthermore, cytokines influence the polarization of macrophages in the TME toward the M1 phenotype, which is proinflammatory and antitumor, further supporting tumor elimination [22].

### 3.3. Currently Approved CAR-T Products and Their Clinical Indications

Kymriah (tisagenlecleucel) is a genetically engineered autologous T-cell immunotherapy that targets CD19 [23]. The drug was approved by the EMA in 2014 and by the FDA in 2017 for the treatment of relapsed and refractory ALL in young adults and pediatric patients. It is also used in the treatment of DLBCL and relapsed/refractory follicular lymphoma (FL) [24].

Tecartus (brexucabtagene autoleucel) is a medication used to treat mantle cell lymphoma in adults when the cancer has come back after two or more prior therapies, and for ALL in people aged 26 years or older when the cancer has come back or has not responded to previous treatments. It was approved by the EMA in 2019 and by the FDA in 2020 [24,25]. CD19-targeted drug demonstrated efficacy in the Phase I/II ELIANA trial in children, adolescents and young adults under 25 years of age for the treatment of relapsed or refractory B-ALL [26,27].

Breyanzi (lisocabtagene maraleucel) is a CD19-targeted, genetically engineered T-cell immunotherapy. It was approved by the FDA in 2021 for use in adult patients with large B-cell lymphoma (LBCL), DLBCL (including DLBCL arising from indolent lymphoma), high-grade large B-cell lymphoma, PMBCL, and stage 3B FL, when resistance to first-line chemoimmunotherapy has occurred or when relapse after first-line therapy has occurred within 12 months. Additionally, when there is ineligibility for hematopoietic stem cell transplantation (HSCT) due to the patient’s age or comorbidities, and relapse/refractoriness of the disease after the second and subsequent lines of treatment [28]. The drug is also used in relapsed and refractory chronic lymphocytic leukemia (CLL) or small lymphocytic lymphoma (SLL), and in the treatment of mantle cell lymphoma (MCL). The EMA approved Breyanzi for use in 2022 [29].

Abecma (idecabtagene Vicleucel) is a drug approved for marketing by the EMA and FDA since 2021. It is primarily used in patients with relapsed and refractory multiple myeloma after two or more lines of treatment, including treatment with an anti-CD38 monoclonal antibody, a proteasome inhibitor, and immunomodulatory agents. Patients with disease progression during the most recent therapy are also included in the treatment group [30]. This therapy is based on a CD-19-negative CAR-T cell and works through antibodies directed against the B-cell maturation antigen (BCMA) [31].

Carvykti (Ciltacabtagene Autoleucel) is a BCMA-targeted CAR-T therapy. It is primarily used in adults with relapsed/refractory multiple myeloma who have previously received one therapy with an immunomodulatory drug and a proteasome inhibitor, and in those who have not responded to lenalidomide. Additionally, the drug will benefit patients whose disease has progressed since their last treatment. The drug was approved by the EMA and FDA in 2022 [32]. Ciltacabtagene Autoleucel demonstrated superior efficacy and survival compared to Idecabtagene Vicleucel in the treatment of relapsed/refractory multiple myeloma [33].

Yescarta (axicabtagene ciloleucel) is a CAR-T therapy targeting the CD19 antigen. It was approved by the FDA in October 2017 for adult patients with relapsed or refractory DLBCL after at least two lines of systemic therapy. The indication also includes primary mediastinal B-cell lymphoma (PMBCL) and FL. In 2018, the drug was also approved by the EMA, and in 2022, the indications were expanded to include second-line treatment for DLBCL and high-grade B-cell lymphoma (HGBL) [34]. The ZUMA-7 study demonstrated the superiority of axi-cel over standard chemotherapy and autologous hematopoietic stem cell transplantation in this group of patients, confirming its clinical value [35].

Aucatzyl (obecabtagen autoleucel, obe-cel) is the latest CD19-targeted CAR-T therapy developed for adult B-ALL patients. The FDA approved it in November 2024, and the EMA issued a conditional marketing authorization in July 2025 [36]. The drug is intended for patients over 26 years of age with relapsed or refractory B-ALL, in whom previous treatments have failed [37]. Clinical trial results indicate a high complete response rate and a favorable safety profile, making Aucatzyl a breakthrough therapeutic option in this particularly difficult-to-treat group of cancers.

### 3.4. Clinical Significance in Practice

A phase I study was conducted, enrolling 23 patients, including 14 with hepatocellular carcinoma (HCC), 7 with pancreatic cancer, and 2 with colorectal cancer [38]. Patients with CD133-positive metastatic disease who had received at least two prior systemic therapies were eligible. Various doses of CAR-T cells, ranging from 0.5 to 2 × 10^6^/kg body weight, were studied. Three patients (13%) achieved a partial response (PR). Median progression-free survival (PFS) in patients with HCC was 7 months, and in all patients, 5 months. Fourteen patients achieved disease stabilization (SD) from 9 weeks to 15.7 months. The 3-month disease control rate (DCR) was 65.2%, and the 6-month DCR was 30.4%. Tumor remission was achieved in 9 patients, and 21 did not develop new metastatic lesions during the study. Patients tolerated the therapy well, with a decrease in hemoglobin and platelets that spontaneously resolved within a week, and hyperbilirubinemia in 3 patients with pre-existing biliary stenosis. Importantly, the antitumor capacity of CART-133 cells correlates positively with their ability to secrete cytokines. Higher levels of IFN-γ, TNF-α, and interleukin-6 (IL-6) were detected in patients who achieved PFS > 8 months [38].

Another phase 1 study included 15 patients with metasoline-expressing tumors—pancreatic ductal carcinoma (n = 5), ovarian adenocarcinoma (n = 5), and pleural mesothelioma (n = 5)—resistant to chemotherapy [39]. Lentivirally transduced CAR-T cell therapy directed against metasoline (CART—meso) was used. These cells were administered at 1–3 × 10^7^ or 10^8^/m^2^ of body weight, with or without 1.5 g/m^2^ of cyclophosphamide, to improve the persistence and efficacy of CAR-T cells (immunoreductive therapy). The best result in this study was disease stabilization in 11 patients—no tumor regression. One 51-year-old woman diagnosed with ovarian cancer 4 years before enrollment in therapy experienced a 26% tumor reduction, but this did not meet RECIST 1.1 criteria. The median PFS was 2.1 months. Cyclophosphamide increased the early expansion of meso-CART cells, but did not prolong their presence. Meso-CART DNA was found in 7 of 10 tumor cell samples, indicating that these cells had entered the tumor. The therapy was generally well tolerated by patients, with no side effects typical of CAR-T, such as cytokine storm or neurotoxicity. Only one patient, who did not receive lymphodepletion, developed sepsis [39].

Another phase I study was conducted in six patients with chemotherapy-resistant pancreatic ductal adenoid carcinoma (PDAC). As in the previous study, patients received CART-meso three times weekly for three weeks. Disease stabilization occurred in two patients, with PFS of 3.8 and 5.4 months, respectively. Metabolically active tumor volume (MAV) was also monitored, stabilizing in three patients and decreasing by 69.2% in one. The therapy was well tolerated by the patients [40].

The Liu et al. study is also a phase I trial that enrolled 16 patients with metastatic pancreatic cancer and epidermal growth factor receptor (EGFR) expression >50% [41]. Thirteen of the 16 patients had previously received chemotherapy with gemcitabine, nab-paclitaxel, fluorouracil, leucovorin, irinotecan, and oxaliplatin (FOLFIRINOX), radiotherapy, or surgery. Patients received co-administering nab-paclitaxel with cyclophosphamide and one to three cycles of EGFR-CART over a 6-month period, with a median CAR -T of 3.48 × 10^6^ cells/kg. Ultimately, 14 patients were available for follow-up. Four patients achieved PR lasting 2–4 months, eight patients demonstrated SD, and the DCR was 85.7%. Median PFS was 3 months, and overall survival (OS) was 4.9 months. CAR-T cells persisted in the blood for approximately 1 month. Importantly, immunohistochemical studies revealed a decrease in EGFR expression in liver tumors and numerous CD3+ T lymphocyte infiltrates in the remission sites [41]. The most serious adverse events were stomatitis, fever, nausea, and fatigue. Additionally, lung parenchymal changes and pleural effusions were detected in two patients. Adverse events are summarized in Table 1.

Another phase I study included CAR-T therapy directed against human epidermal growth factor receptor 2 (HER-2) in patients with advanced biliary tract cancer (n = 6) and PDAC (n = 5) with HER-2 expression >50% [42]. Patients had previously been treated with multiple chemotherapy and/or radiotherapy. They received one cycle of CART-HER2 cells ranging from 0.35 × 10^6^ to 1.0 × 10^7^ cells/kg. Some patients also received cyclophosphamide and nab-paclitaxel before CAR-T therapy. One PR and 5 SD were achieved, and the median PFS was 4.8 months and lasted for 4.5 months. One patient had two metastatic lesions in the liver hilum at the time of enrollment. Four weeks after the first cycle of CAR-T, positron emission tomography coupled with computed tomography (PET-CT) showed disappearance of one metastatic lesion and a decrease in the standardized uptake value of the second lesion from 6.5 to 4.7. The therapy was generally well tolerated by patients, with lymphopenia being the most common finding [42].

The final phase I study cited is a trial involving CAR-T cells retargeted to caludin 18.2 (CLDN18.2), which demonstrated efficacy in the treatment of gastric cancer in preclinical studies [43]. Thirty-seven patients with advanced, refractory gastrointestinal cancers who had CLDN18.2 confirmed in their tumor tissues were enrolled in the study. One element of the therapeutic protocol was so-called bridging therapy, which was administered during CAR-T cell production (which lasted an average of 27 days). The goal of this therapy was to temporarily inhibit cancer progression. One of the drugs used during this period was nab-paclitaxel, an albumin-based paclitaxel complex, which was administered to approximately 32% of patients. Furthermore, nab-paclitaxel was also used as a component of the FNC conditioning regimen (fludarabine, cyclophosphamide, nab-paclitaxel), which was designed to suppress the patient’s immune system and improve CAR-T cell penetration and efficacy. The study used a single, low dose of nab-paclitaxel (100 mg), which was significantly lower than in standard chemotherapy [43]. The study results demonstrated promising efficacy of CT041 therapy. In the entire group of patients (n = 37), the objective response rate (ORR) was 48.6%, and the DCR was 73.0%. The median PFS was 3.7 months, and the 6-month OS was 80.1%. In patients with gastric or gastroesophageal junction cancer (n = 28), the ORR was 57.1%, the DCR was 75%, and the median PFS was 4.2 months. In the subgroup of gastric cancer patients who received the standard dose of 2.5 × 10^8^ cells and had received at least two prior lines of therapy, an ORR of 61.1% and a DCR of 83.3%. The median PFS in this group was 5.6 months, and the 6-month durability of response was 57.1%. The treatment was well tolerated. The most frequently observed adverse events were transient hematologic toxicities typical of conditioning therapy: leukopenia (83.8%), neutropenia (67.6%), and anemia (40.5%). No patient experienced cytokine release syndrome (CRS) above grade 2. Mild CRS (grade 1–2) occurred in 94.6% of patients, but these were easily managed, most often with tocilizumab or glucocorticoids. No neurotoxicity or treatment-related deaths were reported. One patient experienced serious gastrointestinal bleeding (grade 4) associated with rapid tumor shrinkage, and three other patients experienced gastrointestinal complications (grade 3). One patient experienced anaphylaxis after the second infusion, which resolved after treatment [43]. Table 2 summarizes the studies included in the review.

### 3.5. Future Directions and Strategies to Overcome Limitations of CAR-T Therapy in Solid Tumors

Although CAR-T therapy has transformed the treatment of hematological malignancies, its application in solid tumors is still limited by a number of biological and technical obstacles. One of the major challenges is inefficient migration of CAR-T cells into tumor tissue, caused by abnormal vasculature and stromal barriers. To address this, engineered CAR-T cells expressing chemokine receptors complementary to tumor-secreted ligands have been developed to improve trafficking, while loco-regional delivery strategies—such as intrapleural, intraperitoneal, or intracranial infusion—have been tested to bypass systemic distribution hurdles and enhance intratumoral accumulation [44].

Another fundamental obstacle is the highly immunosuppressive tumor microenvironment (TME), which is rich in inhibitory cytokines such as TGF-β and IL-10 and molecules such as programmed death-ligand 1 (PD-L1) and cytotoxic T-lymphocyte associated protein 4 (CTLA-4) that downregulate T cell activity. To counteract this, CAR-T therapy is increasingly combined with immune checkpoint inhibitors, while intrinsic modifications of the cells themselves are also under investigation [45]. These include genetic disruption of inhibitory receptors, the introduction of dominant-negative constructs, or the development of “switch receptors” that convert inhibitory signals into activating ones, thereby restoring effector function in hostile conditions. In parallel, fourth-generation CAR-T cells, also known as TRUCKs, are designed to secrete cytokines such as IL-12 or IL-18 directly within the tumor site, which not only sustains CAR-T activity but also recruits and activates other immune cells, reshaping the TME into a proinflammatory environment [46].

Sustained clinical benefit also depends on the long-term persistence of CAR-T cells, which has proven difficult to achieve in solid tumors. Current strategies therefore focus on optimizing the ex vivo manufacturing process by selecting less differentiated T cell subsets with stem-like or central memory phenotypes, reducing culture times, and supplementing cultures with IL-7 and IL-15 instead of IL-2 to preserve proliferative capacity [47]. At the same time, refinement of CAR design is ongoing, with hybrid costimulatory domains and modified signaling motifs being tested to balance effector potency with long-term survival.

The heterogeneity of tumor antigens poses an additional challenge, as solid tumors can downregulate or lose targeted antigens, leading to immune escape. To overcome this, dual-targeting CAR-T cells capable of recognizing two distinct tumor-associated antigens have been designed, as well as logic-gated systems in which activation requires simultaneous antigen recognition or is suppressed in the presence of healthy-tissue markers [48]. Other approaches target ligands of innate immune receptors such as natural killer group 2 member D (NKG2D), which are broadly expressed in stressed tumor environments, or stromal and vascular elements that are common across many cancer types [49].

Finally, safety remains a critical concern due to the risk of on-target, off-tumor toxicity. To mitigate this, researchers are exploring transient CAR expression through mRNA electroporation, suicide switches that allow for pharmacologic elimination of the infused cells, and “switchable” CAR-T platforms that remain inactive until an adaptor molecule is administered, thereby enabling precise temporal and spatial control over therapeutic activity [50]. Looking ahead, it is likely that the most effective products will combine several of these strategies into a single, multifunctional design

## 4. Discussion

CAR-T therapy is a topic of interest for oncologists worldwide. It is common knowledge that CAR-T therapy has been approved primarily for hematological malignancies, but due to its effectiveness, scientists are exploring other possible applications.

All the clinical trials included were phase I trials—they assess the safety, tolerability, overall feasibility, and technical feasibility of CAR-T therapy for non-hematological cancers. Not all were designed equally, as some used cyclophosphamide to suppress patients’ immune systems, while others, such as the CART-CD133 trial, did not provide such data. Some patients had already received more than one line of treatment, and some had not previously received any. This significantly limits the reliability of the therapy, as it is unclear whether the improved PFS is due to CAR-T activity itself or to other factors, such as CAR-T’s effect on the tumor microenvironment after multiple chemotherapy regimens. Moreover, in one study, sepsis occurred in a patient who did NOT receive lymphodepletion, suggesting that some patients did not receive any conditioning, and its absence may increase the risk of adverse reactions or reduce CAR-T efficacy.

Unlike hematological malignancies, which typically exhibit stable expression of the CD19 antigen, solid tumors are heterogeneous in terms of HER2, EGFR, or CLDN18.2 expression. Furthermore, these antigens may also be present on healthy tissues, raising the risk of on-target, off-tumor toxicity. In the study by Liu et al., the authors also mention heterogeneity in response, which may be due to HER2 receptors. Furthermore, the TME often contains physical barriers, such as dense stroma, suppressor cells—regulatory T cells, tumor-associated macrophages (TAMs), myeloid-derived suppressor cells (MDSCs), and immunosuppressive factors that inhibit CAR-T function.

In the HER2 study, a conditioning regimen containing nab-paclitaxel and cyclophosphamide was previously used before CART-HER infusion, as it has been reported that nab-paclitaxel can deplete desmoplastic stroma and increase tumor vascularization, which may increase CART-HER’s reach of tumor cells [42]. However, a biopsy was not performed to confirm this finding.

Adverse events were mild—fatigue, diarrhea, back pain, and anemia. There were no severe side effects typical of this therapy, with the only severe event being the aforementioned sepsis. However, it is unknown whether these significantly impacted patients’ daily functioning, so further studies should address the patients’ daily well-being. Common side effects typical of CAR-T, such as grade 3–4 CRS or neurotoxicity (ICANS), were not observed. This may be due to limited tumor penetration or reduced CAR-T expansion in vivo, which raises questions about their efficacy. In light of the presented facts, CAR-T therapy is a last-resort therapy; patients were treated with this therapy after several lines of unsuccessful treatment. It would be worthwhile to focus in the future on using CAR-T as a first- or second-phase treatment to avoid exposing patients to such extensive chemotherapy and radiotherapy. Consequently, it would help avoid cachexia, hair loss, which is particularly important for women, and many other potentially life-threatening side effects caused by these therapies. Patients might also better tolerate CAR-T therapy.

In the analysis of studies on solid tumors, adverse events following CAR-T therapy were common, benign or excisional. The most common were fever, lymphopenia, and nausea, while more severe events, such as discharge from the ventricles or transient elevations in transaminases, were sporadic. In contrast, in hematological malignancies (e.g., ALL or DLBCL), the most common and characteristic toxicities are CRS and ICANS, which occur in patients and can reach grade 3–4 in up to 10–20% of treated patients [51]. Therefore, while in hematological settings, the safety of therapy is focused on controlling severe CRS and neurotoxicity, in solid tumors, which are more important, local effects and the resulting “on-target/off-tumor” effects of antigenic impact in the management sense.

To effectively treat CAR-T therapy, it is essential to find appropriate molecular targets to overcome tumor heterogeneity and on-target and off-tumor issues, ensure the persistence of active CAR-T cells, and target and eliminate tumor immunosuppressive cells, including Treg lymphocytes, TAMs, and MDSCs, by combining CAR-T cells with antibodies/drugs that reduce the number of these cells or by generating CAR-T cells that directly target antigens expressed on these cells. Recently published studies provide a new approach to overcoming the immunosuppressive effects of adenosine in the tumor microenvironment, which, through activation of the A2A receptor (A2AR), strongly inhibits the activity of CAR-T cells. The research team proposed a modification of CAR-T cells consisting in the expression of the A1 receptor (A1R), which acts antagonistically to A2AR. Initial experiments showed that constitutive expression of A1R enhances the effector functions of CAR-T cells, but at the same time limits their persistence and long-term expansion in vivo. To address this problem, a system for locally targeted A1R expression was designed, dependent on signals present exclusively within the tumor, thus avoiding undesirable systemic effects. This approach enabled the selective activation of mechanisms supporting the function of cytotoxic CAR-T cells under high adenosine conditions without compromising their overall viability. Gene expression changes associated with this model differed from those observed after A2AR deletion, suggesting that A1R induces a distinct transcriptional pathway [52].

The obtained results support the strategy of targeted expression of CAR-T cell function-enhancing molecules in a context-specific manner, limited to the tumor microenvironment. This approach could provide a universal platform for increasing the efficacy of cell therapy in solid tumors and could be extended to other receptors, cytokines, and immune resistance mechanisms.

One of the key challenges associated with the effectiveness of CAR-T therapy in solid tumors is so-called antigen escape. This phenomenon involves the elimination of tumor cells expressing the target antigen by CAR-T therapy, leading to selection pressure and the growth of a population of tumor cells that lack this antigen or exhibit low expression. In solid tumors, where antigens are often heterogeneously distributed and not always clearly tumor-specific, the risk of selecting therapy-resistant clones is particularly high [53]. Additionally, some cancer cells can actively modulate their cell surface by internalizing or masking antigen, altering glycosylation, expressing inhibitors of costimulatory pathways, or interfering with CAR structure. Strategies to overcome this problem include multispecific CAR-Ts—bivalent or tandem CARs—sequential administration of CAR-Ts with different molecular targets, and combining CAR-T therapy with checkpoint inhibitors (e.g., PD-1/PD-L1), which can support antitumor responses even after partial loss of the target antigen [54].

A crucial, yet often underestimated, aspect of CAR-T therapy is the interpatient variability in clinical response, resulting not only from differences in tumor characteristics but also from individual characteristics of the patient’s immune system. Factors such as the patient’s immune age, the extent of prior cytotoxic treatment, the pool of residual T cells—their diversity, functional status, and the composition of the gut microbiota—can influence the therapy’s effectiveness. Growing evidence indicates that specific bacteria, such as *Faecalibacterium prausnitzii*, can beneficially modulate the response to immunotherapy, while antibiotic therapy prior to CAR-T administration can negatively impact treatment efficacy [55].

A study targeting glypican 3 in HCC is currently underway in patients previously treated with immunotherapy or kinase inhibitors [56]. This is excellent evidence of the development of this therapy and the growing interest in it.

These are just the beginnings of CAR-T use in non-hematological cancers, so the topic remains relatively under-researched. The available studies are only phase 1, so full safety and efficacy results will be available for a long time. Importantly, many studies focus on the use of CAR-T therapy in pancreatic cancer. This is particularly important, as this cancer has one of the highest mortality rates and is most often detected at an advanced stage. This poses a particular challenge for clinicians worldwide; therefore, verifying the effectiveness of CAR-T therapy in pancreatic cancer could revolutionize the treatment of cancer patients and improve their prognosis.

In a meta-analysis, CAR-T in solid tumors has a functional response rate (ORR) of approximately 9% [57]. This would be consistent with the occurrence of complications and clinical disease. In comparison, inhibitory checkpoint inhibitors (ICIs) in cancers have group-specific results—in unselected populations, ORRs typically do not exceed 5–10%, but in MSI-H/dMMR subgroups, they reach 30–40% [58]. Furthermore, inhibitory drug conjugates (ADCs), such as trastuzumab deruxtecan in HER2-positive cancer, have been associated with higher ORR rates (approximately 40%) [59]. In this comparison, CAR-T in tumors exhibited a similar response rate to ICIs, but were significantly lower than ADCs in selected populations.

Overall, CAR-T therapy demonstrates good results and relative safety. It remains unclear how this will play out in long-term follow-up and studies, but science is advancing in the right direction to increase cancer patients’ chances of prolonging their lives. Importantly, and problematically, the realities of affordability may effectively limit its use—the cost of CAR-T therapy can reach several hundred thousand dollars per treatment, raising questions about its accessibility and equity in healthcare. Widespread implementation of CAR-T therapy, particularly outside of hematology, requires consideration of reimbursement, infrastructure costs, and systemic burden. This raises a strong need to revolutionize healthcare policy to support access to such innovative therapies.

Most therapies rely on the patient’s autologous cells, which are time-consuming and expensive. Currently, allogeneic CAR-Ts are being developed, which could be immediately available and significantly cheaper. However, their use is associated with the risk of graft-versus-host disease (GVHD) and the need for additional genetic engineering, such as TCR removal. In the future, this could revolutionize the availability of therapy [60].

Interestingly, on 27 June 2025, the FDA lifted the Risk Evaluation and Mitigation Strategy (REMS) requirement for these products, recognizing that, through clinical experience and labeling updates, the risk of adverse events—such as cytokine release syndrome and neurotoxicity—can be effectively managed without additional procedural requirements [61]. REMS is a system of precautions implemented by the FDA for high-risk drugs, aimed at ensuring their safe use. It included mandatory training for medical personnel, certification of facilities, provision of educational materials for patients, and the requirement for the availability of rescue medications in the event of adverse events. It applied, among others, to CAR-T therapies targeting the CD19 and BCMA antigens, such as Kymriah, Yescarta, Breyanzi, Tecartus, Abecma, and Carvykti [61]. This step demonstrates that scientists are becoming increasingly experienced in conducting this type of therapy, which is why its use in other cancers should be rapidly expanded.

## 5. Conclusions

CAR-T therapy has already revolutionized the treatment landscape for hematological malignancies and represents one of the most important advances in modern oncology. Its translation into the field of solid tumors is progressing more slowly, but the growing body of early-phase clinical trials confirms the feasibility and safety of this therapeutic approach. Although durable responses remain rare, the rapid pace of innovation in cell engineering, manufacturing technologies, and trial design provides a strong rationale for continued development.

Currently, CAR-T therapy in solid tumors remains an experimental therapy, but a growing number of studies and the development of cell engineering technologies offer hope for future breakthroughs in the treatment of these challenging tumors.

## Figures and Tables

**Table 1:** Percentage distribution of the most common adverse events [41].

Adverse Event	Frequency [%]
Lymphocytopenia	88%
Oral mucosal edema	63%
Fever/fatigue	56%
Mucositis oral	43%

**Table 2:** Summary of studies included in the review [33,34,35,36,37,38].

The Title of the Research	Intervention	Patients Number	Construct Type	Lymfodepletion	Type of Study/Phase	Dose	Duration of Follow-Up	Cancers	Endpoints	Results
CD133-directed CAR-T cells for advanced metastasis malignancies: a phase I trial [38]	CART-CD133	23	Anti-CD133 scFv (derived from HW350341.1) + 4-1BB + CD3ζ Lentivirus vector	Not applied.	I phase	0.5 to 2 × 10^6^/kg body weight	24.5 months	HCC, pancreatic cancer, colon cancer	PR PFS SD DCR	PR: 3 patients PFS in HCC: 7months; overall: 5 months SD: 14 patients DCR 3 months 65.2%, DCR 6 months 30.4% Remission: 9 patients
Phase I study of lentiviral-transduced chimeric antigen receptor-modified T cells recognizing mesothelin in advanced solid cancers [39]	CART-meso	15	scFv anty-mesotelin + 4-1BB + CD3ζ; lentiwirus	Some patients received 1.5 g/m^2^ of cyclophosphamide	I phase	1–3 × 10^7^ or 10^8^/m^2^	Tumor assessments at 1, 3, and 6 months after CART-meso infusion, with additional evaluations every 3 months for up to 2 years	Ovarian adenocarcinoma, pancreatic ductal carcinoma, pleural mesothelioma	SD PFS	SD: 11 patients PFS: 2.1 months In 1 patient reduction of tumor mass—26% (did not meet the RECIST 1.1 criteria)
Activity of mesothelin-specific chimeric antigen receptor T cells against pancreatic carcinoma metastases in a phase 1 trial [40]	CART-meso	6	anti-mesothelin ss1 scFv + 4-1BB + CD3ζ; lentivirus vector	Not applied.	I phase	1 to 3 × 10^8^/m^2^ T cells three times weekly	CT imaging—4 months PET/CT imaging—2 months	Pancreatic adenocarcinoma	SD PFS	SD: 2 patients PFS: 3.8 and 5.4 months MAV: stable in 3 patients and reduced in 1 patient
Anti-EGFR chimeric antigen receptor-modified T cells in metastatic pancreatic carcinoma: a phase I clinical trial [41]	CART-EGFR	16	scFv anty-EGFR + 4-1BB + CD3ζ	100–200 mg/m^2^ nab-paklitaksel + 15–35 mg/kg cyklofosfamid	I phase	1.31–8.9 × 10^6^/kg, total 25 cycles	49 months	Pancreatic cancer	PR SD DCR PFS OS	PR lasts 2–4 months: 4 patients SD: 8 patients DCR: 85.7%. PFS median: 3 months, OS median: 4.9 months
Phase I study of chimeric antigen receptor modified T cells in treating HER2-positive advanced biliary tract cancers and pancreatic cancers [42]	CART-HER2	11	scFv anti-HER2 + CD8α + CD3ζ	100–200 mg/m^2^ nab-paklitaksel + 15–35 mg/kg cyklofosfamid	I phase	0.76 × 10^6^ to 1.82 × 10^7^ CAR-T cells/kg body weight in one or more infusions	Not reported.	Cancer of the biliary tract and pancreas	PR SD PFS	PR: 1 patient SD: 5 patients PFS: 4.8 months In 1 patient, disappearance of the metastatic lesion and a decrease in the standardized uptake value of the second lesion from 6.5 to 4.7
Claudin18.2-specific CAR T cells in gastrointestinal cancers: phase 1 trial interim results [43]	CART-CLDN18.2	37	scFv anti-CLDN18.2 + CD3ζ + 4-1BB	Not applied.	I phase	89 patients were dosed with 2.5 × 10^8^, six with 3.75 × 10^8^ and three with 5.0 × 10^8^ CAR T cells	Median: 32.4 months	Stomach cancer and gastric-esophageal junction cancer, pancreatic cancer	ORR DCR PFS OS	ORR: 48.6% DCR: 73.0% PFS: 3.7 months OS: 6 months: 80.1%

## Data Availability

No new data were created or analyzed in this study. Data sharing is not applicable to this article.

## Abbreviations

The following abbreviations are used in this manuscript:

ALL	Acute Lymphoblastic Leukemia
BCMA	B-Cell Maturation Antigen
CAR	Chimeric Antigen Receptor
CAR-T	Chimeric Antigen Receptor T-cell
Cas9	CRISPR-associated protein 9
CLDN18.2	Claudin 18.2
CRS	Cytokine Release Syndrome
CRISPR	Clustered Regularly Interspaced Short Palindromic Repeats
CTLA-4	Cytotoxic T-lymphocyte Associated Protein 4
CTLs	Cytotoxic T Lymphocytes
DCR	Disease Control Rate
DD	Death Domain
DISC	Death-Inducing Signaling Complex
DLBCL	Diffuse Large B-Cell Lymphoma
EGFR	Epidermal growth Factor Receptor
EMA	European Medicines Agency
FADD	Fas-Associated Death Domain Protein
FDA	Food and Drug Administration
FL	Follicular Lymphoma
FNC	Fludarabine, Cyclophosphamide, Nab-paclitaxel
GVHD	Graft-versus-host Disease
HCC	Hepatocellular Carcinoma
HER-2	Human Epidermal Growth Factor Receptor 2
HGBL	High-Grade B-cell Lymphoma
HSCT	Hematopoietic Stem Cell Transplantation
ICANS	Immune Effector Cell-associated Neurotoxicity Syndrome
IFN-γ	Interferon Gamma
IL-6	Interleukin 6
IS	Immunological Synapse
ITAMs	Immunoreceptor Tyrosine-based Activation Motifs
LBCL	Large B-cell Lymphoma
LFA-1	Lymphocyte Function-associated Antigen-1
MAV	Metabolically Active Tumor Volume
MCL	Mantle Cell Lymphoma
MDSCs	Myeloid-Derived Suppressor Cells
NKG2D	Natural Killer Group 2 Member D
OS	Overall Survival
ORR	Objective Response Rate
PDAC	Pancreatic Ductal Adenocarcinoma
PD-L1	Programmed Death-Ligand 1
PET-CT	Positron Emission Tomography Coupled With Computed Tomography
PFS	Progression-Free Survival
PMBCL	Primary Mediastinal B-cell Lymphoma
PR	Partial Response
REMS	Risk Evaluation and Mitigation Strategy
SD	Stable Disease
SLL	Small Lymphocytic Lymphoma
SMACs	Supramolecular Activation Clusters
TAM	Tumor-associated Macrophages
T-cell	Lymphocyte T
TCR/CD3	T-cell receptor/CD3 complex
TNF-α	Tumor Necrosis Factor alpha
TRUCK	T cells Redirected for Antigen-Unrestricted Cytokine-initiated Killing
TME	Tumor Microenvironment
VHH	Variable Domain Of An Antibody Heavy Chain
